# Application of Octacalcium Phosphate with an Innovative Household‐scale Defluoridator Prototype and Behavioral Determinants of its Adoption in Rural Communities of the East African Rift Valley

**DOI:** 10.1002/ieam.4262

**Published:** 2020-05-01

**Authors:** Alfredo Idini, Franco Frau, Luciano Gutierrez, Elisabetta Dore, Giuseppe Nocella, Giorgio Ghiglieri

**Affiliations:** ^1^ Department of Chemical and Geological Sciences University of Cagliari Cagliari Italy; ^2^ Department of Agricultural Sciences and Desertification Research Centre University of Sassari Sassari Italy; ^3^ School of Agriculture, Policy and Development, University of Reading Reading United Kingdom

**Keywords:** Octacalcium Phosphate (OCP), Fluoride contamination of groundwater, Defluoridation method, RANAS behavioral model, Fluorosis

## Abstract

Natural fluoride contamination of drinking water is a serious issue that affects several countries of the world. Its negative health impact is well documented in the East African Rift Valley, where water consumption with fluoride ($F^{-}$) concentration greater than 1.5 mg/L can cause fluorosis to people. Within the framework of the European Union (EU) Horizon 2020 FLOWERED project, we first designed an effective defluoridation device based on innovative application of octacalcium phosphate (OCP) and then explored its acceptance within rural communities. The prototype (FLOWERED Defluoridator Device [FDD]) essentially is composed of a 20‐L tank and a recirculating pump that guarantees the interaction between water and OCP. The device is powered by a car battery for a fixed pumping working time using a fixed amount of OCP for every defluoridation cycle. The results of tests performed in the rural areas of Tanzania show that a standardized use of the prototype can lower the dissolved $F^{-}$ from an initial concentration of 21 mg/L to below the World Health Organization (WHO) drinkable limit of 1.5 mg/L in 2 h without secondary negative effects on water quality. The approximate cost of this device is around US$220, whereas that of OCP is about $0.03/L of treated water. As with any device, acceptance requires a behavioral change on behalf of rural communities that needed to be investigated. To this end, we piloted a survey to explore how psychological and socioeconomic factors influence the consumption of fluoride‐free water. Results show that the adoption of FDD and OCP is more appealing to members of the rural communities who are willing to pay more and have a high consumption of water. Moreover, we suggest that given the low level of knowledge about fluorosis diseases, the government should introduce educational programs to make rural communities aware of the negative health consequences. *Integr Environ Assess Manag* 2020;16:856–870. © 2020 The Authors. *Integrated Environmental Assessment and Management* published by Wiley Periodicals LLC on behalf of Society of Environmental Toxicology & Chemistry (SETAC)

## KEY POINTS

- Octacalcium phosphate (OCP) can effectively remove fluoride (F^−^) from natural waters of the East African Rift Valley (EARV) through its transformation into fluorapatite (FAP).
- A new device for the treatment of fluoride‐polluted waters based on OCP has been presented: the FLOWERED Defluoridator Device (FDD). It has been tested and the results show that the FDD can be effectively applied in rural contexts.
- A standardized method for fluoride‐rich water treatment has been settled up: in 2 h of working time the FDD with 80 g of OCP can treat 20 L of natural water with F^−^ concentration up to 21 mg/L.
- From socio‐economic analysis emerges that, given the poor knowledge of the risk of dental and skeletal fluorosis, the EARV governments should introduce educational programs to make rural communities aware of the negative health consequence of these diseases.

## INTRODUCTION

Geogenic contamination of fluoride ($F^{-}$) in groundwater above the World Health Organization (WHO) limit of 1.5 mg/L (WHO 2017) is an issue that affects the health of about 260 million people in many countries worldwide (Amini et al. 2008; Biswas et al. 2017; Akuno et al. 2019; Chowdhury et al. 2019). Fluoride‐rich drinking water is a primary source of dietary $F^{-}$ that can overcome the tolerable upper intake level (UL) of 10 mg/d for adults and from 0.7 to 2.2 mg/d for infants and children (US Institute of Medicine 2006). High $F^{-}$ intake causes different adverse health effects on teeth and bones, such as dental and skeletal fluorosis, and toxic effects on nonskeletal tissues, such as the nervous system, cardiovascular system, liver, kidney, reproductive system, thyroid, and the progeny, which have been extensively studied in the last few years (Ozsvath 2009; Barbier et al. 2010; Wei et al. 2019). Endemic fluorosis, both skeletal and nonskeletal, is still diagnosed in many countries (Tanzania, Kenya, Ethiopia, India, China, etc.) (Hunter et al. 2010) especially where rural communities are exposed to fluoride‐rich groundwater.

Within the framework of the Horizon 2020 European‐funded “de‐FLuoridation technologies for imprOving quality of WatEr and agRo‐animal products along the East African Rift Valley in the context of aDaptation to climate change” (FLOWERED) project (www.floweredproject.org), we focused our research on the development and acceptance of an innovative defluoridation method that can be effective for rural communities, which is mandatory to achieve one of the Sustainable Development Goals of United Nations, specifically Goal 6: Clean water and sanitation (https://sustainabledevelopment.un.org/sdg6).

An effective and frugal defluoridation method should be 1) low cost, to make even the poorest part of the population able to access to drinkable water; 2) low tech based, easy to use and distribute, to reach the population regardless of where people live; 3) highly efficient, to avoid waste of water resource; and 4) free of collateral effects on the overall water quality. Despite the great number of technologies and materials successfully tested at a laboratory scale, the challenge to find an effective and reliable in‐situ method is still open (Ayoob et al. 2008; Yadav et al. 2018). There are several reasons why the defluoridation of water in rural contexts is an open challenge. Up to now, this seems to be determined by the lack of technology transfer to a feasible method in terms of overall cost and power supply (e.g., reverse osmosis), complexity of the method (e.g., electrocoagulation), cost of sorbent supply and management (e.g., most of synthetic compounds), or low $F^{-}$ removal capacity (e.g., bone char [BC] and many natural materials) (Khairnar et al. 2015; Mumtaz et al. 2015; Velazquez‐Jimenez et al. 2015). Furthermore, the acceptance of any new defluoridation method on behalf of rural communities is critical for policymakers and other stakeholders if they want to facilitate the adoption of the technology (Sorlini et al. 2011; Datturi et al. 2015). Therefore, the objectives of the present study are to conduct in‐situ defluoridation tests with a new device loaded with octacalcium phosphate (OCP; Ca_8_(HPO_4_)_2_(PO_4_)_4_ ∙ 5H_2_O), and to explore rural communities' acceptance of this new technology. The abovementioned tests were performed in northern Tanzania to assess the applicability of this new technology in the same environmental conditions faced by potential adopters of these rural populations. The starting point for the development of the technology was based on the absorption and accumulation mechanisms of $F^{-}$ in human tissues. The inorganic part of human bones and teeth consists of bioapatite, a mineral with a general formula similar to hydroxyapatite (HAP; Ca_5–x_(PO_4_)_3–y_OH_1–z_, where the most common substitutions are *x* = Na^+^, Mg^2+^; *y* = CO_3_^2−^, HPO_4_^2−^; *z* = $F^{-}$, Cl^−^) (Wopenka and Pasteris 2005). The formation of bioapatite is subsequent to the formation of its precursor: the OCP (Markovic and Chow 2010; Carino et al. 2018). One of the processes of bioaccumulation of $F^{-}$ into the hard tissue is the formation of fluorapatite (FAP; Ca_5_(PO_4_)_3_F) instead of bioapatite (Everett 2011). The underpinning key concept of the method for water defluoridation is to transfer the mechanism of $F^{-}$ absorption by the human body to a defluoridator device based on the same mineralogical principle. A previous research paper showed that OCP has empirical $F^{-}$ removal capacity of 25.7 mg/g; the water treatment lasts a few hours and is free of collateral effects (Idini et al. 2019). To meet the requirement “easy to use” targeted for rural communities of the East African Rift Valley (EARV), a standardization of the defluoridation procedure and use of the prototype named “FLOWERED Defluoridator Device” (FDD) is presented. Importantly, the purpose of the standardized use is to guarantee the defluoridation of water regardless of a defined range of the initial $F^{-}$ concentration.

However, the availability of a new efficient defluoridation method that allows EARV rural communities to drink fluoride‐free water may not be enough to increase the consumption of safe water. Switching from untreated drinking water to safe defluoridated water can be challenging for individuals living in these rural communities. This is because the use of defluoridated water and the acceptance of any new device such as the OCP and FDD can be influenced by socioeconomic and psychological factors that may affect the adoption of the new healthy drinking behavior. Thus, we piloted a survey where the Risk, Attitude, Norm, Ability, Self‐regulation (RANAS) model (Mosler 2012) was applied to explore how these factors can influence the consumption of fluoride‐free water in EARV rural communities.

## STUDY AREA

### Tanzanian scenario: Fluoride, fluorosis, and its mitigation

**Figure 1:**
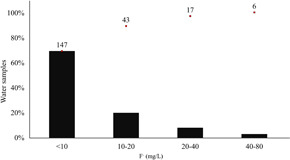
Range and distribution of F^−^ contamination in 213 samples of natural water of EARV (Giaciri and Davies 1993; Tekle‐Haimanot et al. 2006; Ghiglieri et al. 2012; Olaka et al. 2016; Malago et al. 2017). Red squares represent the number of samples in each range. EARV = East African Rift Valley.

In the last decades, several kinds of research have clarified that the geogenic origin of $F^{-}$ contamination is strictly linked to the water interaction with the volcanic lithology of the EARV and that high $F^{-}$ concentration is associated to sodium‐bicarbonate water facies (Giaciri and Davies 1993; Gizaw 1996; D'Alessandro 2006; Rango et al. 2009; Ghiglieri et al. 2012; Olaka et al. 2016). It is important to emphasize that this hydrogeochemical feature is the same worldwide in the fluorotic belts (Chowdhury et al. 2019). Collecting 213 data from cited literature of EARV fluorotic areas (Giaciri and Davies 1993; Tekle‐Haimanot et al. 2006; Ghiglieri et al. 2012; Olaka et al. 2016; Malago et al. 2017), it can be estimated that the range of geogenic contamination of waters by $F^{-}$ is under 40 mg/L in 97% of water sources used for human consumption (Figure 1).

**Figure 2:**
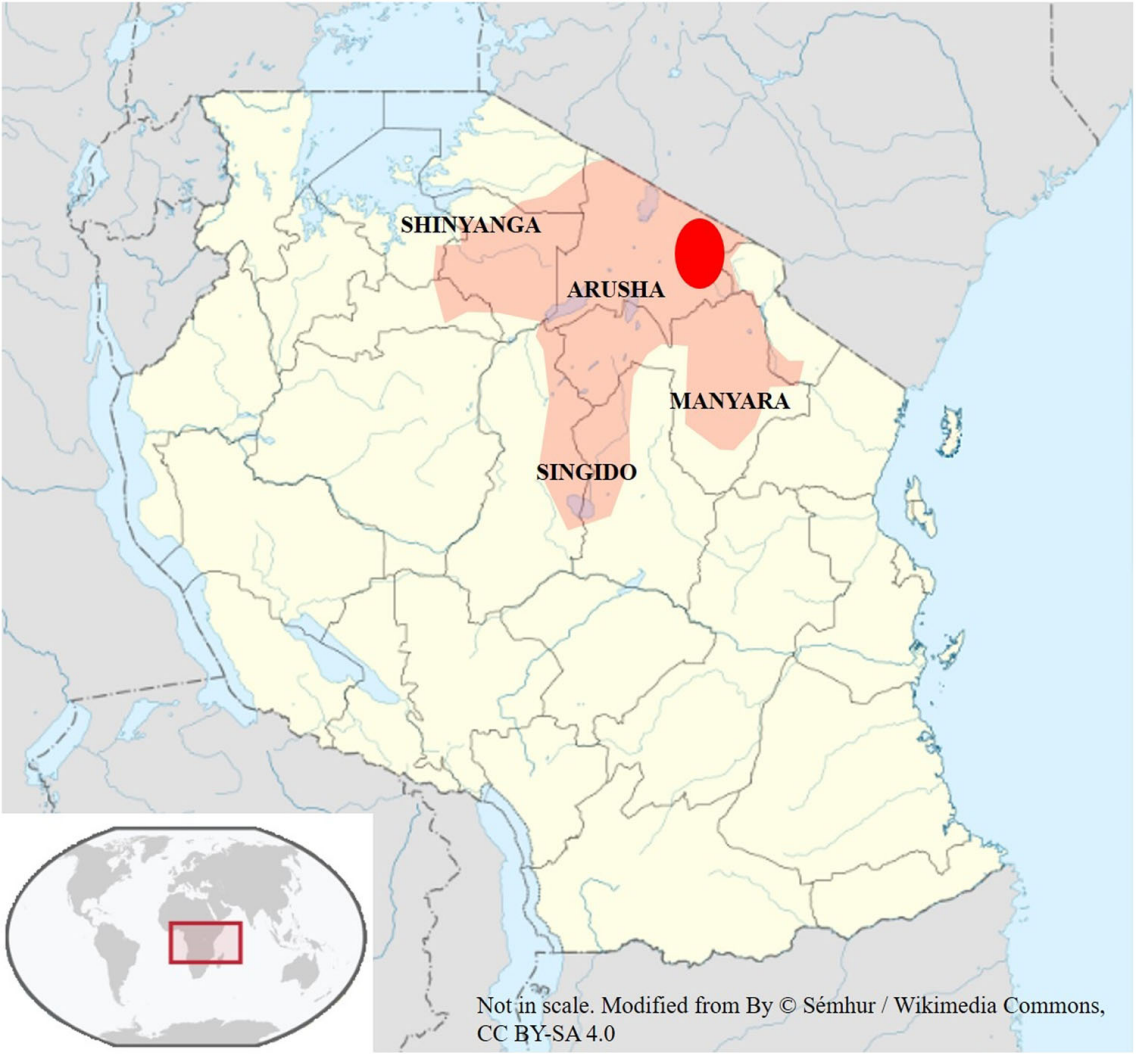
Sketch map of fluorotic regions of Tanzania, extrapolated from Malago et al. (2017). The red circle indicates the area of defluoridation tests and interviews.

Tanzanian occurrence of $F^{-}$ rich groundwater and its relationship with fluorosis has been documented since 1944 (Grech and Latham 1964). Current Tanzanian regulation and guideline for drinking water set the permissible $F^{-}$ limit to 4 mg/L (Tanzania Bureau of Standard 2018). In 2010 the Ministry of Water and Irrigation (MoWI) issued the National Water Quality Management and Pollution Control Strategy 2010 (SMEC 2010) in which it explained that about 10.5 million people are exposed to drinking water with $F^{-}$ concentration above the WHO limit; this population lives in the regions of Arusha, Manyara, Singido, and Shinyanga (Figure 2). Up to now, not only the rural communities have been exposed to fluorosis but also the urban population, as happens in the urban area of Arusha (Chacha et al. 2018). The MoWI document highlights that, despite successful research (Mbabaye et al. 2017), outcomes and development efforts have not been widely implemented, and many people still consume water containing $F^{-}$ levels above the healthy limit.

## MATERIALS AND METHODS

### Synthesis of octacalcium phosphate (OCP)

The OCP was synthesized in 2 steps (Supplemental Data Table S1): 1) synthetic brushite (dicalcium phosphate dihydrate [DCPD]; CaHPO_4_ ∙ 2H_2_O) was synthesized at room temperature adding 0.366 moles of both H_3_PO_4_ and CaCO_3_ in 2 L of tap water (Supplemental Data Table S2) acidified at pH 1.5 with HCl. After precipitation, the DCPD was recovered through filtration and dried at 40 °C; 2) 1.2 g of DCPD was added to 500 mL of tap water, and the solution was then heated in a stove at 60 °C for 65 h; the solid was recovered through filtration and dried at room temperature. The synthesized OCP is a white powder with particle size in the range of 14 to 125 µm, determined using a mesh sieves. All the reagents were of analytical grade (Carlo Erba reagents ACS‐for analysis) and used without further purification. The synthesis procedure was carried out in the laboratories of the Department of Chemical and Geological Sciences (DCGS), University of Cagliari, Italy.

### FLOWERED Defluoridator Device (FDD)

**Figure 3:**
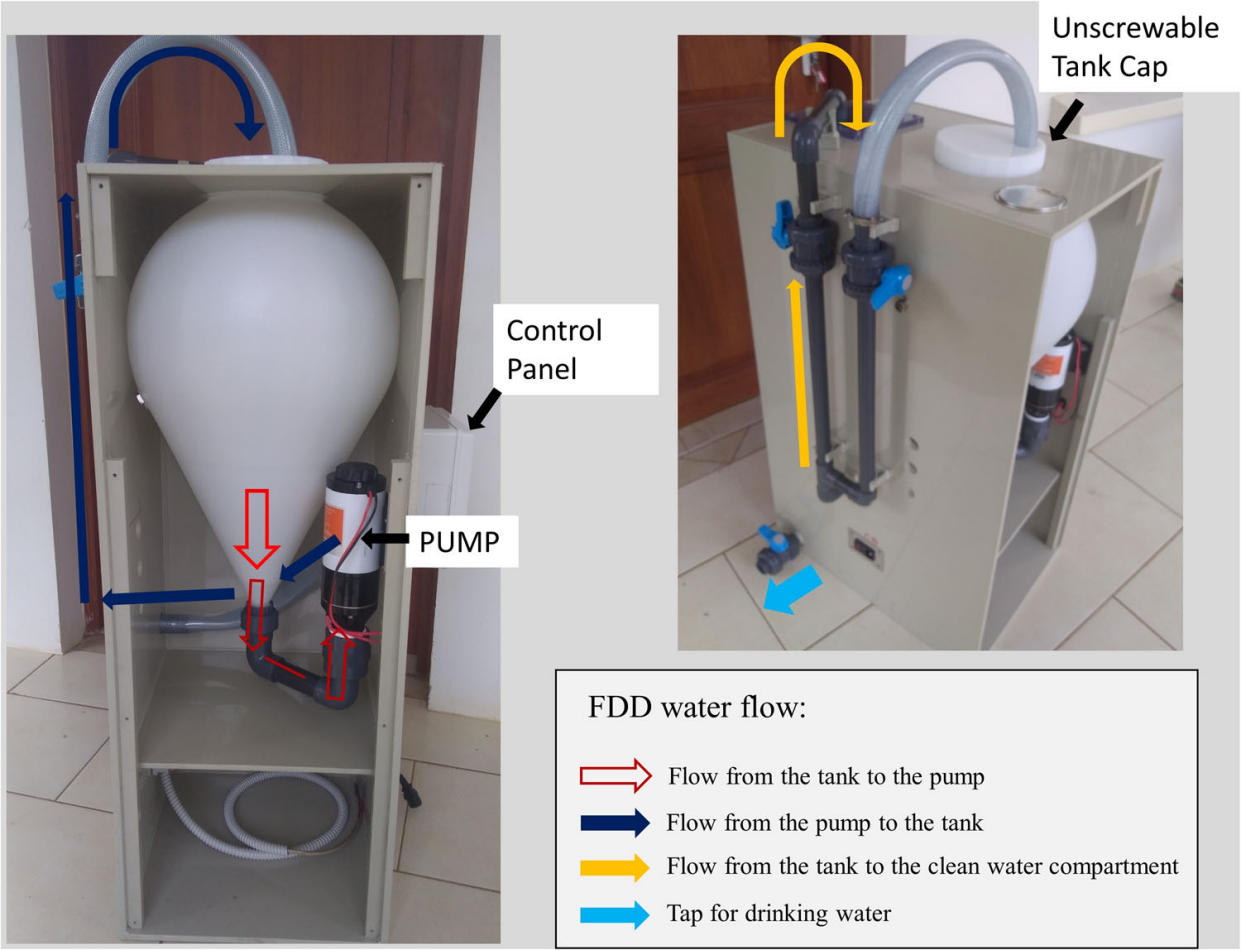
The FLOWERED Defluoridator Device (FDD) and its recirculating water circuit.

The FDD is a conceptual reproduction of a laboratory batch experiment designed to meet the rural household application. It is composed of a 25‐L polyethylene terephthalate (PET) drop‐shape tank (where OCP and water interact) (Figure 3). An electric recirculating‐macerator pump (model LIGHTEU‐SEAFLO 12 V −12 A × h, nominal water volume flow rate 45 L/min; 32.2 × 15.3 × 10.9 cm; weight 2.6 kg) is necessary to maintain the mixing between OCP and water. The pump is powered by a car battery (locally bought, model FB 38B19L‐MF, voltage 12 V and capacity 28 A × 5 h–35 A × 20 h, Furukawa Battery CO Ltd) to guarantee a recirculating flow rate of 22 L/min constant for 3 h. The FDD battery can be recharged by different power supplies (e.g., solar panel, power generator, electric grid). An elastic nylon net, located in a second compartment (clean water compartment), is necessary to separate exhausted sorbent from water and to collect the treated water. The pump, tank, and compartment for treated water are connected by pipes and faucets and are assembled in a 100 × 43 × 55−cm plastic case. The total weight is 23 kg. The FDD has been designed and assembled by the FLOWERED technological partner Hydro Technical Engineering (HTE) s.r.l. (Verona, Italy).

### Defluoridation field tests

**Figure 4:**
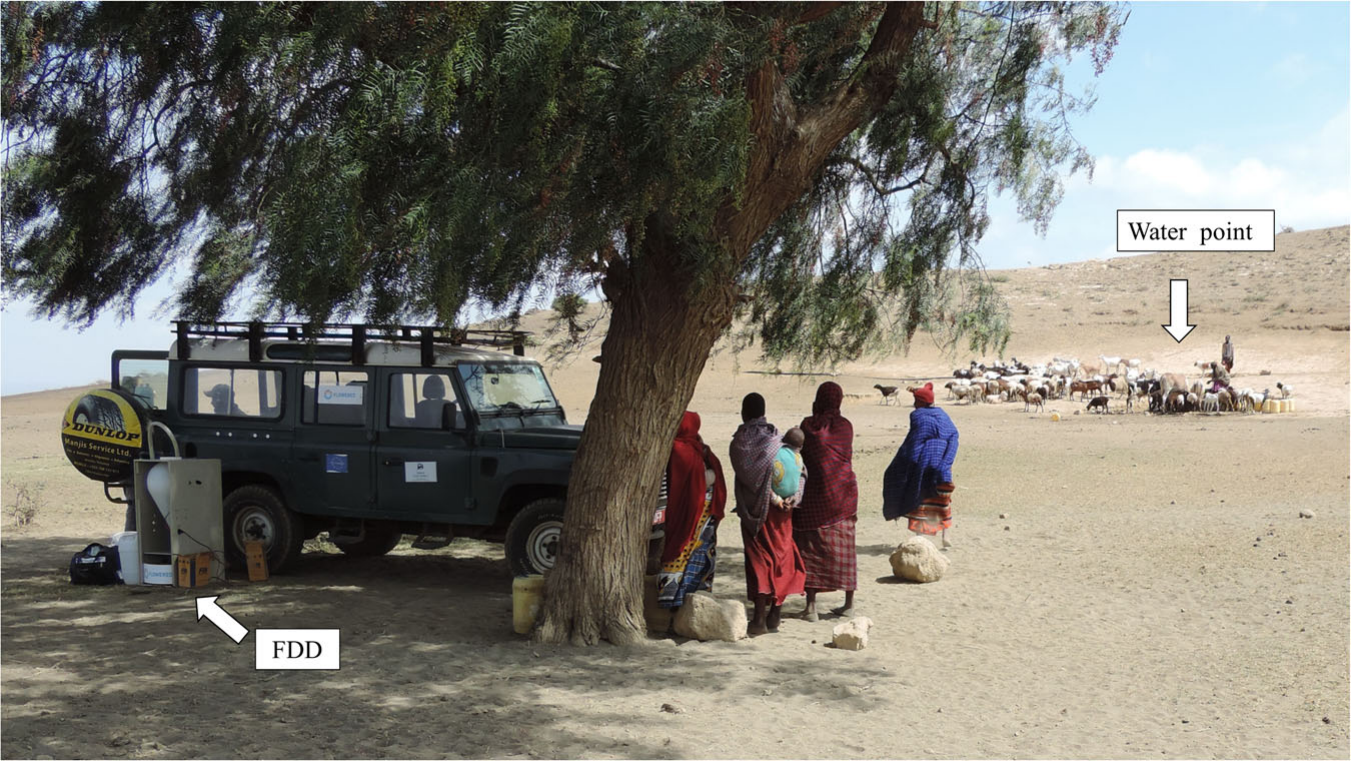
Site of defluoridation experiment in rural areas around Mount Meru, Northern Tanzania. On the left side the FLOWERED Defluoridator Device (FDD) during defluoridation test and behind a typical water point used for human and animal consumptions.

The defluoridation field tests (Figure 4; Supplemental Data Figures S1, S2, S3, and S4) were performed in different areas using 3 natural waters (labeled BUL for Bule Bule spring, NGO for Maji ya Chai spring, and KYU for Ngarenanyuki borehole) with different initial $F^{-}$ concentrations ($F^{-}$ i.c.) (Table 1). The reason for choosing those areas was because the geogenic contamination of $F^{-}$ in the Arusha Region is well documented. Besides, the selected water points are representative of the range of $F^{-}$ concentration of the water the rural population drinks. Another important criterion was the finding that in those areas a defluoridation method is not available at all.

**Table 1:** Water points and their initial F^−^ concentration used for defluoridation tests

Label sample	Location	Coordinates		Waterpoint type	F^–^ i.c. (mg/L)
BUL	Bule Bule spring, Uwiro village, Tanzania	3°10′39.66″S	36°51′10.68″E	Diffuse spring	8.4
NGO	Maji ya Chai spring, Ngurdoto village, Tanzania	3°17′36.60″S	36°52′43.32″E	Catch spring	20.9
KYU	Ngarenanyuki, secondary school, Tanzania	3°15′5.84″S	36°54′47.54″E	Borehole	37.2

i.c. = initial concentration.

All the defluoridation field tests were performed as follows: 20 L of natural water was collected directly from the spring or borehole, poured directly with 80 g of OCP into the FDD through the unscrewable cap of the tank, and the pump was turned on. The concentration of $F^{-}$ in the water was measured during the FDD working time. After the $F^{-}$ concentration had decreased below the WHO drinkable limit, the faucet of the flow from the pump to the tank was closed and the faucet of the flow from the pump to the treated water compartment was opened. In the second compartment, an elastic nylon net recovered the exhausted OCP powder. At the end of the process, the treated water was collected from the tap (Figure 3) and the exhausted OCP was recovered from the recovery compartment. The FDD was carefully washed with the same water as the next experiment. Considering the OCP empirical removal $F^{-}$ capacity of 25.7 mg/g, obtained during lab‐scale batch experiments performed with the OCP synthesized with the same method (Idini et al. 2019), and the aim of obtaining the defluoridation in about 2 h of FDD working time, different dosages of OCP (solid‐to‐liquid ratio [S/L] equals to 1, 2, and 4 g/L) were tested using NGO water ($F^{-}$ i.c. = 20.9 mg/L). The choice of 2 h as optimal FDD working time will be discussed in *Defluoridation tests*.

### Chemical analyses and mineralogical characterization

At the field experiment sites, the pH and the $F^{-}$ concentrations of both untreated and treated water were determined, respectively, using a portable pH meter (Hanna) and a potentiometer (HQ30d Portable meter, HACH) equipped with an ion‐selective $F^{-}$ electrode (IntelliCALTM ISEF121, HACH). Fluoride measurements were performed by adding the TISAB III solution (Total Ionic Strength Adjustment Buffer, concentrated for $F^{-}$ analyses, HACH), in the recommended volume ratio 1:5 (v/v) between TISAB III and standard or sample solutions, to buffer the pH and avoid the interference of metal complexes. For each defluoridation test, portions of untreated water and water recovered at the end of reaction time were collected and stored in a polyethylene (PE) bottle at 4 °C to perform chemical analysis. Before chemical analysis, all water samples were filtered (0.4‐μm pore size, OlimPeak, Teknokroma). The concentrations of major ions were determined through ion chromatography (IC; Dionex ICS3000) and inductively coupled plasma optical emission spectroscopy (ICP‐OES; ARL Fisons 3520), and the total alkalinity was determined with the Gran method.

The mineralogical characterization of OCP, before and after the tests, was performed collecting X‐ray diffraction (XRD) patterns in the 3.5° to 55° 2θ angular range on an automated PANalytical X'pert Pro diffractometer, with Ni‐filtered Cu‐K*α*_1_ radiation (*λ* = 1.54060 Å), operating at 40 kV and 40 mA, using the X'Celerator detector. The software used for mineralogical identification and analysis was X'Pert HighScore Plus version 2.1b (Degen et al. 2014). The percentage of crystallinity of the samples recovered after the experiments, defined by the intensity ratio (I_net_) of the diffraction peaks on the sum of all measured intensities, was calculated according to Equation 1 subtracting from the total intensity (I_tot_) the constant background intensity (Bgr_const_) of OCP crystals 125 to 150 µm in size, used as external standard:

(1) $$\text{Crystallinity}\;[\%]=100\times {\Sigma \;I}_{\text{net}}/({\Sigma \;I}_{\text{tot}}-{\text{Bgr}}_{\text{const}}).$$

Semiquantitative mass fraction calculation [%w/w] was performed from diffraction data by normalized reference intensity ratio (RIR) algorithm (Chung 1974), using the RIR value of OCP and FAP from the International Centre of Diffraction Data (ICDD; http://www.icdd.com/).

### The socioeconomic survey

To achieve the stated objective, a questionnaire was developed and piloted in rural communities of North Tanzania using the RANAS model (Mosler 2012). The RANAS model has been applied in a few similar studies (Huber et al. 2012; Huber and Mosler 2013), where researchers have attempted to evaluate how the psychological elements of this conceptual framework can influence the use of devices and/or the adoption of healthy drinking behavior, that is, defluoridated drinking and cooking water.

In the present study, the pilot questionnaire was divided into 3 sections, and information about questions, items, and values of measurement scales are reported in Supplemental Data Table S3. The first section (Supplemental Data Table S3) elicited information about water drinking habits and the 5 elements of the RANAS. The 9 risk items captured perceived vulnerability and perceived severity and factual knowledge of individuals toward dental and skeletal fluorosis. The 2 vulnerability items measured individuals' perceived probability of getting these 2 diseases, whereas severity items assessed the negative consequences of these diseases on health. The 5 factual knowledge items tested whether respondents knew how to prevent these diseases correctly by applying simple precautions (boiling water, brushing teeth, taking medicine, and drinking milk). Five attitude items evaluated both positive aspects of drinking defluoridated water concerning good health, reducing medical expenses, better taste, and feeling happy, and negative aspects in terms of time management. Three normative items elicited information about what respondents think other community members will do (descriptive norm), what other people think they should do (injunctive norm), and their commitment toward healthy drinking behavior (personal norm) if the rural community had the possibility of using these new OCP and FFD. The ability item measured the personal capacity of an individual to carry out healthy drinking behavior, whereas the 5 self‐regulation items gathered information about plans regarding the use of these devices in terms of daily routine, disaster, and commitment. Other than vulnerability items evaluated on a 5‐point scale ranging from very unlikely to very likely, factual knowledge is captured with yes/no answers, and all the other components of the RANAS model were measured on a Likert scale ranging from completely disagree to completely agree.

The second section collected information on the willingness to pay where participants were prompted with a hypothetical market scenario and asked to state the maximum amount that they were willing to pay for OCP. It was assessed following an open‐ended format.

The third section gathered information about sociodemographic characteristics of respondents such as gender, age, education, assets of individuals, and household amenities such as main sources of drinking water and minutes necessary to collect drinking water on foot from home.

The pilot study was preceded by a training workshop where researchers in collaboration with the OIKOS East Africa, a nongovernmental organization in Tanzania, explained to interviewers the aim of the study, providing them with information about $F^{-}$, dental and skeletal fluorosis diseases, and use of FDD whose model was shown and tested in the same area where this study was conducted. Interviewers were also trained on how to collect data using tablets. The pilot survey was administered between May and June 2019, conducting 100 random face‐to‐face interviews in 7 villages near the city of Arusha: Oldonyowas, Uwiro, Lemanda, Engutukoit, Lemongo, Losinoni Juua, and Losinoni Kati (Figure 2 and Supplemental Data Figure S2). These hundred participants were water consumption decision makers of 5000 households living in an area of the world where collecting information is very challenging. The questionnaire was translated from English into Swahili by OIKOS East Africa and during data collection, the interviewers were supervised by 2 technicians from this nongovernmental agency.

Data analysis was conducted using STATA 15 (StataCorp 2017). For the multi‐item components of the RANAS model, a Cronbach's alpha test of reliability was performed to evaluate their internal consistency as average aggregate scores. A logistic regression (2) was estimated to determine how the probability of consuming defluoridated water (CFW) is influenced by the sociodemographic characteristics of participants and the components of the RANAS models as follows:

(2) $$P({\mathrm{CFW}}_{j}\;|\;X_{1j},\;\text{…},X_{kj})=\frac{1}{1\;+\;e^{-(\beta_{0}+\sum_{i=1}^{k}\beta_{i}X_{ij}+\epsilon_{j})}},$$

where P(*CFW*_*j*_ | *X*_1*j*_, …, *X*_*kj*_) is the conditional probability of consuming defluoridated water (i.e., adoption of healthy drinking and cooking behavior) given the set of psychological and sociodemographic variables (*X*_*ij*_ *i* = 1, …, *k*); *i* is an index ranging from 1 to *k*, indicating the number of variables; *j* is an index ranging from 1 to *N*, indicating the number of individuals; *β*_0_ is the intercept; *β*_*i*_ are the parameters; and $\epsilon_{j}$ is the error term. The adequacy of the model was evaluated by the pseudo coefficient of determination *R* ^2^ and the goodness‐of‐fit test for logistic regression proposed by Hosmer and Lemeshow (1980).

## RESULTS

### Defluoridation tests

Three different OCP dosages were tested with NGO water ($F^{-}$ i.c. = 20.9 mg/L): 20, 40, and 80 g of OCP (S/L respectively equals of 1, 2, and 4 g/L in the tests NGOa, NGOb and NGOc). The results show that when starting with 20 L of natural water with 20.9 mg/L F^−^, the FDD can reach the WHO limit of 1.5 mg/L F^−^ respectively in 12, 6, and 2 h as a function of the increasing OCP dosage (Table 2).

**Table 2:** Effect of different solid‐to‐liquid ratio (S/L = 1, 2, and 4 g/L respectively in the tests NGOa, NGOb and NGOc) on time needed to decrease the F^−^ water concentration below the WHO limit of 1.5 mg/L

Label	OCP dosage (g/L)	F^−^ (mg/L)		Time (h)
		Initial	Final	
NGOa	1	20.9	1.28	12
NGOc	2	20.9	1.40	6
NGOb	4	20.9	1.32	2

NGO = Maji ya Chai spring, Tanzania; OCP = octacalcium phosphate; WHO = World Health Organization.

Considering the removal capacity of OCP of 25.7 mg/g, as mentioned in the *Introduction*, the theoretical minimum OCP dosage for effectively treating the NGO water is 0.82 g/L. This theoretical calculation is confirmed by the result of the NGOa test which uses a slightly higher dosage of 1 g/L. Increasing the OCP dosage from 1 to 4 g/L decreases the time required to reach the drinkable limit from 12 to 2 h (Table 2).

**Figure 5:**
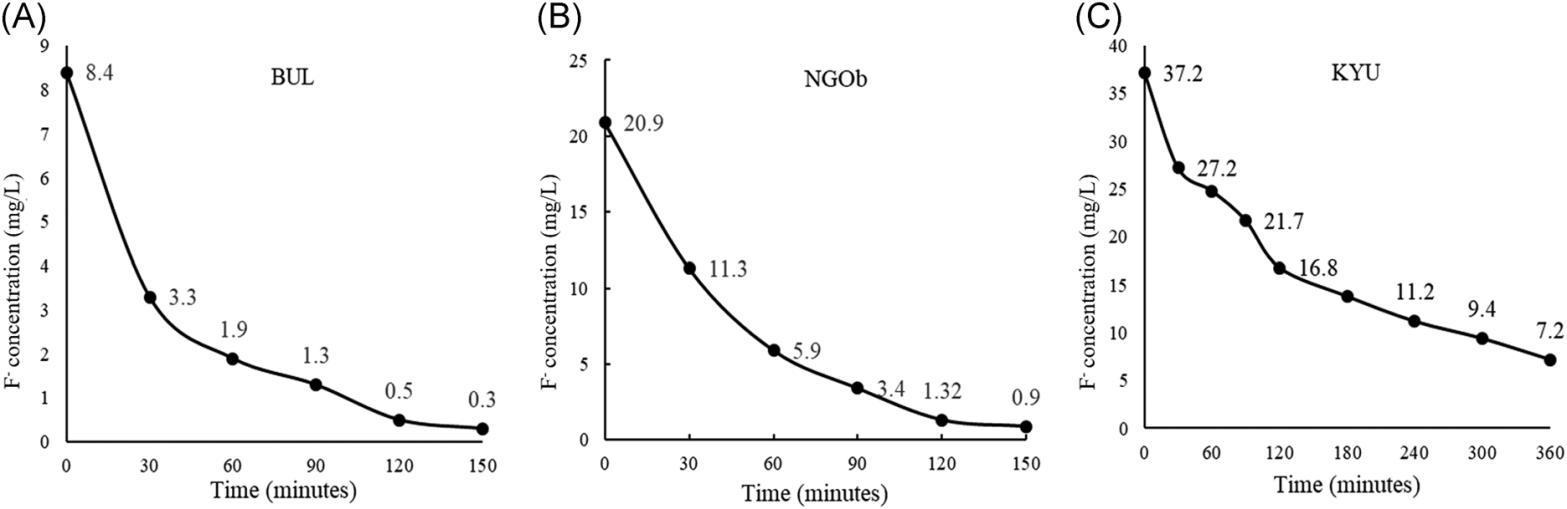
The plots show the removal trend of dissolved F^–^ by 80 g of OCP in 20 L (S/L = 4 g/L) of 3 different natural waters (**A** = BUL, **B** = NGOb, **C** = KYU) in different step times and different initial F concentrations. BUL = Bule Bule spring, Tanzania; KYU = Ngarenanyuki borehole, Tanzania; NGO = Maji ya Chai spring, Tanzania; OCP = octacalcium phosphate.

The dosage of 4 g/L of OCP was tested also with BUL ($F^{-}$ i.c. = 8.4 mg/L) and KYU waters ($F^{-}$ i.c. = 37.2 mg/L) (Figure 5). The results show that in 2 h FDD charged with 4 g/L of OCP decreases the dissolved $F^{-}$ concentration below the WHO limit (1.5 mg/L) from the BUL water (Figure 5A). In the KYU test, after 6 h of treatment, the $F^{-}$ concentration decreases from 37.2 to 7.2 mg/L, but as shown in Figure 5C, the reaction did not reach the equilibrium.

The main variations on NGO water after defluoridation treatment with different dosages of OCP (Table 3) indicate that the pH goes close to the neutrality; moreover, the decrease of HCO_3_^−^ from 427 to 299 mg/L and the increase of P^5+^ up to 46.5 mg/L are observed. The increase of P^5+^ is explainable by the higher molar Ca‐to‐P ratio in FAP (1.67) to the starting molar Ca‐to‐P ratio in OCP (1.33). The small increase of Na^+^, K^+^, SO_4_^2−^, and Cl^−^ concentrations in treated water may be related to the tap water used for OCP synthesis. The behavior of Ca^2+^ can be linked to the OCP dissolution and precipitation of FAP: after 2 h (NGOb sample) FAP is not fully precipitated, whereas after 6 h (NGOc sample), where Ca^2+^ is less than the initial concentration, the precipitation of FAP is complete.

**Table 3:** The compositional difference of natural water after defluoridation treatment with different dosage of OCP and different contact time

Sample	pH	Ca^2+^ mg/L	Mg^2+^ mg/L	Na^+^ mg/L	K^+^ mg/L	P^5+^ mg/L	SO_4_^2−^ mg/L	Cl^−^ mg/L	HCO_3_^−^ mg/L	F^−^ mg/L
NGO	8.45	4.2	<1.5	176	24.4	0.3	27.3	19.6	427	20.9
NGOb (4 g/L, 2 h)	6.96	8.1	<1.5	191	27.5	46.5	34.8	28.5	299	0.9
NGOc (2 g/L, 6 h)	7.18	2.5	<1.5	194	28.4	37.1	33.9	27.3	329	1.4

NGO = Maji ya Chai spring, Tanzania; OCP = octacalcium phosphate.

### Effect on solid phase

**Figure 6:**
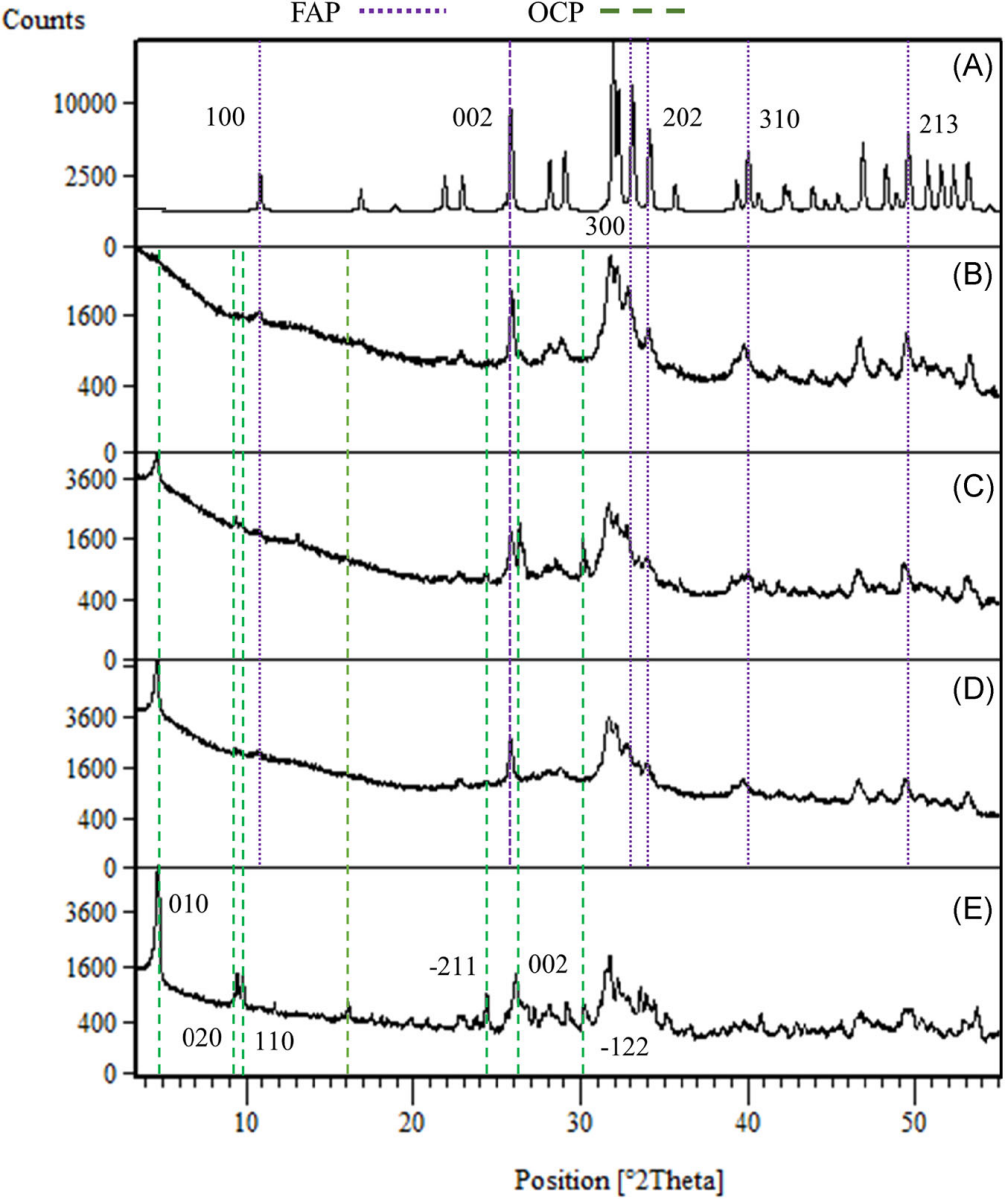
XRD patterns of standard FAP (reference ICSD pattern n. 00‐015‐0876) (**A**); sorbent recovered after KYU experiment (**B**); sorbent after NGOb experiment (**C**); sorbent after BUL experiment (**D**); OCP before experiment (**E**). BUL = Bule Bule spring, Tanzania; FAP = fluorapatite; ICSD = Inorganic Crystal Structure Database; KYU = Ngarenanyuki borehole, Tanzania; NGO = Maji ya Chai spring, Tanzania; OCP = octacalcium phosphate.

The XRD patterns of sorbent (Figure 6), before and after the tests, point out the transformation of OCP into FAP:

(3) $${\text{Ca}}_{8}{\left({\text{HPO}}_{4}\right)}_{2}{\left({\text{PO}}_{4}\right)}_{4}\;\cdot \;5H_{2}O_{\text{OCP}}+1.6F^{-}\to 1.6{\text{Ca}}_{5}{\left({\text{PO}}_{4}\right)}_{3}F_{\text{FAP}}\;+\;1.2{\text{HPO}}_{4}^{\;2-}+5H_{2}O+0.8H^{+},$$

recognizable by its XRD peaks (100) (002) (202) (310) and (213). In the XRD patterns of the solid phase after the NGOb and BUL tests (Figures 6C and 6D) some residual OCP is clearly detectable by the presence of the characteristic peak (010) and/or twin peaks (020) (110) and (002) (−122) (−211). The XRD pattern from KYU test ($F^{-}$ i.c. = 37.2 mg/L) shows more similar shape and peak position with FAP (Figure 6B) and the OCP characteristic peaks (010) (020) (110) are not visible but, because both OCP and FAP share similar XRD patterns in the range 20° to 40° 2θ (Iijima et al. 1996), the differences observed in the experimental XRD patterns in this angular range (Supplemental Data Figure S3) provide important information on secondary peaks and permit the OCP identification even from the KYU test. Phases other than OCP or FAP were not detected.

The crystallinity of the solids recovered after the tests decreases by 30% in BUL after 2.5 h, 31% in NGO after 2.5 h, and 22% in KYU after 6 h. Semiquantitative mass fractions analysis (%w/w) of detected phases calculated by RIR indicates a positive relationship with removed $F^{-}$ from solution (Table 4) and OCP‐to‐FAP ratio in the solids recovered after the tests. In all tests reported in Table 4, characterized by an S/L ratio of 4 g/L, the amount of residual unreacted OCP is higher than the amount of formed FAP; this is because it is necessary to use more OCP than the stoichiometric amount to reduce the treatment from 12 to 2 h.

**Table 4:** Relationship between F^−^ removed by 80 g of OCP from 20 L of the solution and FAP in the solid phases after defluoridation tests determined by RIR of crystalline phases

	Solutions			Solids	
Sample	F^−^ i.c. (mg/L)	F^−^ f.c. (mg/L)	F^−^ removed (mg)	% w/w OCP	% w/w FAP
BUL	8.4	0.3	162	89	11
NGOb	20.9	0.9	400	82	18
KYU	37.2	7.2	600	68	32

BUL = Bule Bule spring, Tanzania; FAP = fluorapatite; F^−^ f.c. = fluoride concentration at the end of experiments; F^−^ i.c. = fluoride initial concentration; KYU = Ngarenanyuki borehole, Tanzania; NGO = Maji ya Chai spring, Tanzania; OCP = octacalcium phosphate; RIR = reference intensity ratio.

### Preliminary results of the socioeconomic study

#### Descriptive statistics

Results of the pilot study show that 74% of *N* respondents (*N* = 100) were female with an average age of 44.3 y (standard deviation [SD] = 14.5, median [Md] = 46.5) and an average household size of 5.6 (SD = 2.7, Md = 5). Regarding education, 35% of respondents were illiterate, 44% had completed primary education, and 21% had a level of education higher than primary school. Furthermore, 72% of respondents reported fetching a daily average of 2.5 (SD = 1.46, Md = 2) buckets (20 L) of drinking and cooking water, and only 52% declared to have consumed defluoridated water.

Table 5 presents the descriptive statistics of the variables used in the logistic regression analysis, the Cronbach's alpha values of the average aggregate scores of the RANAS components, and the results of the backward logistic regression analysis. Findings indicate that the Cronbach's alpha test of the reliability of the multi‐item components of the RANAS model is very good for the vulnerability (0.96), good for severity (0.73) and attitude (0.80), and barely acceptable for self‐regulation (0.54). On the average, participants have a very low level of knowledge (Md = 0.48, SD = 0.25), feel moderately vulnerable (Md = 1.33; SD = 1.20) to dental and skeletal fluorosis, and believe that their health would be affected severely by these diseases (Md = 3.33, SD = 0.43). Furthermore, descriptive, injunctive, and personal norms mean values range from 1.19 to 1.33 and thus emphasize how important these norms are for EARV communities. Participants also scored very high on ability and self‐regulation, and therefore they feel confident using and managing this new FDD. The average distance in minutes between the main source of water and respondents' homes was within 30 min, while the average willingness to pay for consuming 20 L of fluoride‐free water was 89.92 Tanzanian shillings (TZS), equal to US$0.04 in January 2020, with high variability.

**Table 5:** Descriptive statistics and results of the logistic regression analysis on the consumption of fluoride‐free water (*N* = 100)

		Descriptive statistics regression results							
Factors/Covariates		Range	M	SD	α	*β*	SE *β*	*Z*	*P* > |*Z*|
Risk beliefs	Vulnerability	[0−4]	1.335	1.204	0.962	−0.859**	0.363	−2.360	0.018
	Severity	[0−4]	3.330	0.428	0.735	1.920*	0.913	2.100	0.036
	Factual knowledge	[0−1]	0.484	0.253		*−0.651*	1.775	−0.290	0.772
Attitudinal beliefs		[−2 to 2]	0.868	0.340	0.796	0.978	1.031	0.950	0.343
Normative beliefs	Descriptive	[−2 to 2]	1.330	0.513	—	−2.061	1.313	−1.570	0.122
	Injunctive	[−2 to 2]	1.190	0.734	—	−1.358	0.891	−1.520	0.135
	Personal	[−2 to 2]	1.290	0.477	—	2.222*	0.946	2.350	0.017
Ability beliefs		[−2 to 2]	1.230	0.736	—	2.074*	0.998	2.080	0.038
Self‐regulation beliefs		[0−4]	2.964	0.435	0.539	−0.521	1.114	−0.460	0.649
Perceived distance		[0−5]	0.820	0.989	—	*0.970* **	0.358	2.710	0.007
Willingness to pay for fluoride‐free water		Open	188.1	1001.6	—	*0.007* *	0.003	2.280	0.023
Age		Open	44.67	15.39		0.065**	0.024	2.700	0.007
Water consumption (number of 20 L buckets per week)		Open	15.480	9.070		0.122**	0.046	2.660	0.008
Constant						−11.620**	4.218	−2.760	0.006
The fit of the model						Log‐likelihood = −45.779 LR χ (13) = 46.91 (*p* < 0.000) Pseudo *R* ^2^ = 0.339 Hosmer‐Lemeshow χ (8) = 9.66 (*p* = 0.289)			

M stands for the mean values, *α* is the Cronbach (1951) measure, β are the logistic estimates, SE β their standard errors.

*, **Denote a significance value at 5% and 1% significance level respectively.

#### Logistic regression analysis

Results of the logistic regression (right side of Table 5) show that data fit the model quite well. The Hosmer‐Lemeshow's goodness of fit test is not significant and therefore we reject the hypothesis that the model differs significantly from the observed data. Variables used in the model explain 34% of the total variance (pseudo *R* ^2^ = 0.339) in the consumption of defluoridated water, with factual knowledge, attitude, descriptive norm, injunctive norm, and self‐regulation that turned out not to be significant. The significant and positive beta parameters of severity, personal norm, and ability indicate that participants who consume more defluoridated water are those concerned about the negative health consequences of these diseases, feeling strong obligations to adopt the same healthy drinking behavior of their rural community and being confident about the use of the new technology. For example, when all other variables are equal to their means, the severity beta parameter (*β* = 1.92) indicates that a marginal increment of its aggregate score will increase the probability of consuming defluoridated water by about 38%. By the same token, the beta parameter of the personal norm (*β* = 2.22) shows that a marginal increment of its score will increase the probability of consuming defluoridated water by nearly 45%. On the other hand, vulnerability shows a negative relationship with the dependent variable, that is, the higher the perceived likelihood of getting these diseases, the lower the consumption of defluoridated water. Even though the sign of this parameter was expected to be in the opposite direction, this behavior might be explained by the fact that when people feel extremely vulnerable, they do not believe or might not be motivated to adopt healthy drinking behavior. In this case, ceteris paribus, the vulnerability beta parameter (*β* = 1.92) indicates that a marginal increment of its aggregate score will decrease the probability of consuming defluoridated water by about 17%. The walking time of distance (0 indicates less than 14 min, 4 indicates more than 60 min) from the main source of water (*β* = 0.97) and the willingness to pay for fluoride‐free water (*β* = 0.03) had a positive impact on the probability to consume defluoridated water. The first result implies that if the main source of water is distant from the participant's home, the probability of consuming fluoride‐free water is higher than when this is close to them. This factor probably considers the implicit costs associated with the time spent in fetching water. Summing‐up this implicit cost to the actual price reduces the relative price of consuming fluoride‐free water and increases its demand.

Finally, the beta parameters of respondents' age (*β* = 0.06), perceived distance from the main source of water (*β* = 0.97), the weekly consumption of 20 L buckets of water (*β* = 0.12), and willingness to pay for fluoride‐free water (*β* = 0.03) influenced positively the consumption of defluoridated water. Thus, keeping all other variables equal to their means, the probability of adopting healthy drinking behavior increases by 13% when participants are 10 y older, by 19% if perceived walking time of distance increases by 15 min, by about 2.5% for every extra bucket of water consumed, and by 1% for those who are willing to pay 100 TZS more for defluoridated water obtained using the new device.

## DISCUSSION

### Water defluoridation

The experimental data show that FDD charged with 80 g of OCP in 20 L (S/L = 4 g/L) of natural water can reduce the $F^{-}$ content below the WHO limit for drinking water of 1.5 mg/L from initial concentrations of 8.4 mg/L (BUL water) and 20.9 mg/L (NGOb water) (Figures 5A and 5B) in 2 h. With $F^{-}$ i.c. = 20.9 mg/L of NGOc and NGOa tests, the drinkable limit was reached in 6 and 12 h charging FDD with 2 g/L and 1 g/L of OCP, respectively.

In the test with $F^{-}$ i.c. = 37.2 mg/L (KYU) the $F^{-}$ concentration significantly decreases after 6 h of treatment (down to 7.2 mg/L). It is possible to note that, after 6 h of treatment, the equilibrium was not still reached (Figure 5C) and that about 68% w/w of OCP was still unreacted (Table 4). Failure to reach a final $F^{-}$ concentration ($F^{-}$ f.c.) below the drinkable limit of 1.5 mg/L was probably due to power loss of battery that occurred after about 2.5 h of working time, with consequent reduction of the flow to the pump that lowered the stirring rate and thus affected the reaction kinetics between OCP and water. On the other hand, the amount of residual unreacted OCP is always higher than the amount of formed FAP in the tests with S/L of 4 g/L (Table 4), and this is because the $F^{-}$ i.c. in each test is far insufficient to transform all the OCP into FAP according to the stoichiometry of the reaction OCP → FAP (Supplemental Data Table S1). This excess of OCP favors a fast water treatment, a key feature for an effective and frugal defluoridation method. The seeming contradiction between XRD data (Figure 6) and semiquantitative calculation (Table 4) is because OCP tends to easily lose in crystallinity when in contact with water and its (010) peak dramatically decreases in intensity.

According to the Guidelines for Drinking Water (WHO 2017), it is worthwhile to underline that no deleterious collateral effects on the quality of treated water were produced during the defluoridation process; in fact, the solution pH slightly decreases but remains in the range suggested by WHO for drinking water (pH = 6.5−9.5) and the elements listed in Table 4 are within the drinkable limits. Moreover, the increase of dissolved P^5+^ (Table 3), which is not subject to limitations for drinking water (WHO 2017), can be considered a positive effect because it is an essential element for life with recommended dietary reference intake (DRI) of 100 mg/d for infants, 700 mg/d for pregnant women and adults, and 1250 mg/d for children and teenagers (US Institute of Medicine 2006).

### Standardization of the use and procedure: Sorbent dose and optimum FDD working time

In order to meet the requirement for a disclosable method targeted to rural communities, some important considerations must be taken into account. Usually, rural people have a moderate knowledge of the $F^{-}$ concentration in the water they drink daily, which can change in different areas and during the seasons and/or year by year. Without knowing this value, it is impossible to know how much sorbent and/or what contact time between sorbent and water is necessary to effectively defluoridate water. For instance, without a monitoring system, using a column‐like method, it is impossible to know after how many liters of water the sorbent is exhausted and needs to be replaced. For these reasons, taking into account the use in a rural scenario, a standardization of the procedure regardless of the initial $F^{-}$ concentrations is recommended. Also, a reasonable defluoridation time is another important factor. Reducing the sorbent amount per cycle at the minimum (1 g/L) implies to extend the working time of the FDD pump, which means more power consumption and battery wear. After these considerations, we suggest 2 h as an optimum FDD working time because we consider 2 h as a reasonable time for users and for saving the life of the battery. Up to now, the standard parameters are these: FDD loaded with 4 g of OCP per liter (20 L of water, 80 g of OCP), 2 h of mixing with a pump flow of 22 L/min. It is important to emphasize that this estimation is also based on the fact that about 89% of water for human consumption in the EARV has up to 20 mg/L F^−^. Considering the results with $F^{-}$ i.c. = 37.2 mg/L (KYU test) and the OCP removal $F^{-}$ capacity, the next development will be focused on improving the FDD stirring process to guarantee the defluoridation of water with up to 40 mg/L F^−^ applying the above‐reported standardized parameters. Moreover, in case of grid power supply and/or if the $F^{-}$ i.c. is known and can be monitored, the amount of OCP necessary for defluoridation can be lowered to the minimum amount of 1 g/L instead of the standard S/L ratio of 4 g/L.

### FLOWERED Defluoridator Device (FDD)

The key concept of FDD is to replicate a defluoridation batch process that needs mixing and stirring between sorbent (or a chemical reagent for $F^{-}$ precipitation) and water. This means that FDD is not only OCP related but can be loaded with different compounds using sorbent reagent‐related dose and mixing time. Every load of fluoride‐rich water needs a dose of sorbent, and after every defluoridation cycle the exhausted sorbent can be recovered from the dedicated compartment. The FDD could also be used with an adsorbent material if the stirring system would significantly improve the defluoridation kinetics. The power supply through the battery is designed to be charged from different sources, such as solar panel, power generator, power grid, and so on. Finally, the FDD weight (23 kg), similar to the weight of water that can be treated per cycle (20 L), and the size 103 × 43 × 55 cm are targeted for household use.

### Sustainability

#### Production of OCP

The synthesis of OCP requires calcium carbonate and phosphoric acid as sources for Ca^2+^ and P^5+^. Considering industrial grade calcium carbonate and phosphoric acid, the reagent cost of 1 kg of OCP is about = $6 to $8, depending on the Tanzania marketplace. Considering the standardization of the use (4 g/L), 1 kg of OCP can treat 250 L of water, which means a cost of about $0.024 to $0.032/L of defluoridated water. The use of local georesources to synthesize the sorbent material can greatly improve its economic sustainability. Mineral sources of both Ca^2+^ and P^5+^ are already cultivated in Tanzania: Calcium carbonate is exploited for the cement industry from a limestone quarry in the eastern part of Tanzania, from Tanga to Dar Er Salaam (Stewart and Muhegi 1989; Nicholas et al. 2006; Jacob 2019), and P is one of the major elements of the phosphorite rock exploited in the central‐northern part of Tanzania for fertilizer production (Msolla et al. 2005; Szilas et al. 2008). Further research will be focused on the use of local georesources and optimization of the OCP synthesis procedure.

#### Disposal of used sorbent

The solid phase recovered after the standardized defluoridation process is composed of OCP and FAP in variable percentages (Table 4). For the disposal of the OCP–FAP mix, different hypotheses can be formulated, first by using the OCP–FAP mix as a fertilizer in the same way in which natural FAP is already used in Tanzania (Msolla et al. 2005; Szilas et al. 2008). However, a focused study about the interaction of the OCP–FAP mix with soil and $F^{-}$ from irrigation water is mandatory to understand if 1) the OCP–FAP mix can still immobilize $F^{-}$ from soil and irrigation water or 2) the OCP–FAP mix can release $F^{-}$ to the soil and or to the cultivation. The second hypothesis is based on different solubilities of OCP (8 mg/L) and FAP (0.2 mg/L) (Dorozhkin 2012): Adequate washing with water can dissolve only the OCP from the OCP–FAP mix to recover Ca^2+^ and P^5+^ and then restart the synthesis of new OCP. The residual FAP could be used as a source of P^5+^ to produce phosphate fertilizer and/or phosphoric acid (Schrödter et al. 2008; Kongshaug et al. 2014).

#### FLOWERED Defluoridator Device (FDD)

Due to the simpleness of the components of the FDD and the possibility of a multichoice power supply source, the FDD meets the “low‐tech” requirement for a disclosable method targeted for rural areas. Up to now, the overall cost of the FDD is about $220; the most expensive components are the pump (about $60) and the battery (about $60–$80). It is important to consider that the FDD is a prototype, and thus a subsequent industrial scale‐up can improve all the technological aspects (i.e., maintenance aspect, different material instead of a plastic case, etc.) and costs. Theoretically, the FDD is designed to last for as many years as the life of the pump.

### Comparison with other in situ household‐scale defluoridation methods

As mentioned in the *Introduction*, in the last decade more than 800 different sorbent materials with a promising $F^{-}$ removal capacity have been proposed in peer‐reviewed literature. However, because the experimental laboratory conditions may differ greatly from natural field conditions, only the defluoridation methods effectively applied at household scale in rural contexts with natural water will be taken into consideration. Moreover, advanced treatment technologies, such as reverse osmosis, electrodialysis, and membrane distillation, have been excluded from the comparison because they cannot be used at household scale in rural areas of developing countries due to economic and technological limitations (e.g., power consumption, management of sludge water, maintenance cost) (Yadav et al. 2018).

The first defluoridation method applied in a rural context in India was the Nalgonda technique, involving the use of Al salts, lime, and bleaching powder, but it is no longer recommended due to the release of elements (i.e., Al, sulfate) above the drinkable limit into the defluoridated water and to the difficulty in managing the water treatment method (Ingle et al. 2014). Other defluoridation methods already in use at the village scale in some limited rural areas of developing countries are mainly based on BC (Ethiopia, Kenya, Tanzania, Senegal, South Africa, Thailand) (Kut et al. 2016) or activated alumina (India, Sri Lanka) (Fawell et al. 2006). However, both methods require a trained user for the management of the reagent and monitoring the water quality, whereas our main goal was to develop a very simple method that can be used directly by the population at household scale in rural contexts without any specific training. This is a very important aspect because the abandonment of a water treatment method in rural areas is often linked to the impossibility of finding one or more users with the ability and, above all, the will to manage the purification system constantly and correctly.

As far as we know, the only 2 methods already applied at household scale are based on a bucket–column type filter loaded with activated alumina, especially in India, or BC, especially in the EARV. The $F^{-}$ removal capacity (Langmuir Q_max_) of activated alumina is 2.40 mg/g (Ghorai and Pant 2005), whereas for OCP it is 26.8 mg/g (Idini et al. 2019). Moreover, the use of activated alumina, as reported in the literature (George et al. 2010; Jadhav et al. 2015; Yadav et al. 2018), can easily release Al into the treated water above the drinkable limit of 0.1 mg/L due to Al–F^−^ chemical complexes (e.g., AlF^++^, AlF_2_^+^, AlF_3_°, AlF_4_^−^) that are very stable in solution and subtract Al from precipitating as oxide or hydroxide, notably increasing Al solubility, and thus the health risk, given that Al is very toxic. Also, as recognized by the WHO (Fawell et al. 2006), the water treatment with activated alumina requires good operating conditions, mainly the use of optimum pH for the adsorption process and the avoidance of excessive Al dosage (this means that the concentration of $F^{-}$ must be known before starting each treatment to correctly dose the reagent). Because of all these aspects, we have considered activated alumina a nonreliable reagent for household‐scale application according to our goals.

Bone char is a calcium phosphate compound prepared by charring animal bones. The removal mechanism is based mainly on ion exchange between $F^{-}$ and OH^−^ (Alkurdi et al. 2019):

(4) $${\text{Ca}}_{5}{\left({\text{PO}}_{4}\right)}_{3}\text{OH}+F^{-}\to {\text{Ca}}_{5}{\left({\text{PO}}_{4}\right)}_{3}F+{\text{OH}}^{-}.$$

Charring the bones at the optimal temperature of 400 °C, the calculated $F^{-}$ removal capacity (Q_max_) is 3.5 mg/g, whereas the empirical removal capacity using synthetic water with 21 mg/L $F^{-}$ in a laboratory environment decreases to 0.985 mg/g (Mbabaye et al. 2017). That means that 19 g of BC are needed to reach the drinkable $F^{-}$ WHO limit of 1.5 mg/L. For comparison, as reported in Table 2, starting from a $F^{-}$ concentration of 20.9 mg/L, 1 g of OCP is more than enough to reach the drinkable limit. Very similar results about the BC removal capacity were obtained in Senegal (Sorlini et al. 2011), in Kenya (Korir et al. 2009), and in Ethiopia and Tanzania (Dahi 2016), where the BC method was implemented in small rural areas by governmental and nongovernmental organizations such as Nakuru defluoridation company, which is an initiative of the Catholic diocese of Nakuru, Kenya (https://nakurudefluoridation.co.ke/), and Oromo Self‐Help Organization (OSHO) in Wayo Gabriel, Ethiopia. The data reported on these areas indicate that BC can work properly if the water to be treated has $F^{-}$ content less than 8 mg/L (Albertus et al. 2000).

The cost of water treatment by sorbent reagent depends mainly on $F^{-}$ concentration, or equally, how much $F^{-}$ is to be removed from the water, given that the sorbent is consumed during the removal reactions. Because the concentration of $F^{-}$ is highly variable site by site, and even varies during the seasons, it is not possible to compare the cost of 1 L of treated water for different methods, unless a standard $F^{-}$ water concentration is assumed. Considering a removal capacity of 0.9 mg/g for the BC system (Albertus et al. 2000) and an average BC cost of $0.30/kg, the approximate cost to remove 1 g of $F^{-}$ from water is $0.33. This value is very similar to the cost calculated for OCP: Taking into account the empirical removal capacity of 25.7 mg/g and an average OCP cost of $7/kg, the approximate cost to remove 1 g of $F^{-}$ is $0.27.

Moreover, switching to the standardization of the method presented here (*Standardization of the use and procedure: Sorbent dose and optimum FDD working time*), with the advantage that water quality monitoring is not required and can treat the water regardless of its $F^{-}$ concentration up to 21 mg/L, the cost of 20 L of treated water is $0.28.

The cost of US$220 for the FDD is more expensive than the bucket filter, which can range from $19 to $45, depending on the manufacturer. The difference between a batch system, as the FDD, and a bucket–column system, as in the BC method, consists in the use of the sorbent: In a batch system a single dose of the sorbent is poured into the water in every single cycle; in a bucket–column system the water passes through a fixed amount of sorbent, usually 8.5 kg of BC. In order to know when the BC load of 8.5 kg is exhausted, either constant monitoring of $F^{-}$ concentration in raw water, due to its variability in space and time, or periodic monitoring of $F^{-}$ concentration in treated water would be necessary. The monitoring of the bucket–column system at household scale is considered an important limitation for its effective use: The analysis of dissolved $F^{-}$ concentration requires a specific chemical laboratory or expensive portable equipment to be used by a highly trained specialist. Up to now, a reliable, low‐cost $F^{-}$ detection method that can be used in situ by nonexpert users has not been developed, although some effort in this regard is ongoing (López‐Alled et al. 2017).

### Implications of the socioeconomic analysis

Preliminary results of the socioeconomic analysis indicate that respondents feel capable of employing the OCP and FDD regularly, supporting the idea that their use is a relatively easy activity to perform. This belief can facilitate the acceptance of the new device as a simple and efficient method for obtaining and consuming defluoridated drinking and cooking water. The adoption of healthy drinking behavior seems to be more appealing to members of the EARV rural communities, who are willing to pay more for the new technology and have a high consumption of water. However, findings also show that this is an area of the world dominated by a high degree of illiteracy, and people have a very low level of knowledge about dental and skeletal fluorosis. This indicates that the government should introduce educational programs to make EARV rural communities aware of the negative health consequences of these diseases. Educational programs should also motivate people to adopt healthy drinking behavior, making them aware that these new devices can reduce oral intake of water with elevated levels of $F^{-}$. Such approaches might reverse the observed negative relationship between perceived vulnerability and the safe drinking water. Furthermore, because personal norms are so important in the adoption of defluoridated safe water, stakeholders should consider how to change ingrained habits of unsafe water use in the EARV rural community. This could be achieved with demonstration fields having the scope first to unfreeze the bad ingrained habits of drinking untreated water and then changing people's attitudes with information related to how the new technology helps to reduce the risk of getting these diseases. Persuasion could start with older members of rural communities because they appear to be more susceptible to adopting healthy drinking behavior and then be propagated to younger members because of the positive impact of personal norms. The positive impact of moral obligation on the adoption of healthy behavior using a new defluoridator was also found in a study conducted by Huber et al. (2012).

## 1 SUMMARY

The present study employs a multidisciplinary approach to develop a defluoridator prototype for the EARV rural communities and to explore what socioeconomic and psychological factors can facilitate its acceptance within these rural populations. For this purpose, OCP, which has shown effective $F^{-}$ removal capacity in previous batch tests, was used with a new defluoridator prototype, the FDD. The FDD is a new low‐cost and low‐tech device, designed to reproduce, in the field, the defluoridation laboratory process that requires mixing and stirring between the sorbent and the water. The FDD experimentation was carried out in the rural areas of northern Tanzania, at the same environmental conditions of its possible future use by rural populations, using 3 natural fluoride‐rich waters with different $F^{-}$ starting concentrations and testing different sorbent dosage. In agreement with our previous study, the results of the FDD field tests show that the OCP effectively removes the $F^{-}$ from natural water by means of the transformation of OCP into FAP. Based on these results, a standardized use of FDD and dosage of OCP for the defluoridation of natural water has been presented: FDD loaded with 20 L of natural water with $F^{-}$ concentration up to 21 mg/L, 80 g of OCP, and 2 h of FDD working time with constant mixing flow pump of 22 L/min. Further research will be focused on the sustainability of OCP production, using already available local georesources, and its green disposal after use.

Finally, preliminary results of the socioeconomic study are encouraging and support the proposed device as an easy activity to accomplish, as testified by the positive and significant coefficient of the ability variable in the logistic regression. However, from the analysis it emerges that, given the poor knowledge of the risk of dental and skeletal fluorosis, the government should introduce educational programs to make EARV rural communities aware of the negative health consequences of these diseases. Raising knowledge about the danger of consuming untreated water via educational campaigns can improve the consumption of fluoride‐free water in the Rift Valley region. Increasing awareness can also reverse the sense of powerlessness observed for vulnerability in the logistic regression, making members of EARV rural communities more aware that the use of this device can reduce their vulnerability. Thus, the intervention of government agencies and donors seems to be an important aspect to consider mitigating the cost of defluoridation and to ensure access to clean and safe water for all communities as required by Goal 6 of the UN Sustainable Development Goals.

## SUPPLEMENTAL DATA

In the Supplemental Data, readers can find more information about the chemical reaction, behavior factors used in the analysis, and values used for the questionnaire and the map of the defluoridation experiments.

**Figure S1.** Site of defluoridation test and interview in the Uwiro village, Northern Tanzania.

**Figure S2.** Location of water point source used for defluoridation test (dotted circle) and interview (purple line is around the Engutukoit village; red line is around Oldonyowas and Losinoni villages; yellow line is around Uwiro and Lemanda villages; Arusha Region, Northern Tanzania). Image © 2019 Digital Globe‐ Image © 2019 CNES/Airbus.

**Figure S3.** Configuration of the FLOWERED Defluoridator Device (FDD).

**Figure S4.** Step‐by‐step procedure for water treatment with the FLOWERED Defluoridator Device (FDD) and octacalcium phosphate powder (OCP).

**Figure S5.** Difference of XRD pattern between OCP before tests (black pattern), solids collected after KYU (blue pattern) and BUL (red pattern) defluoridation tests and FAP reference ICSD pattern n. 00‐015‐0876 (green lines). In the pattern of KYU experiment, where 600 mg of $F^{-}$ was removed from solution, the residual peak of OCP is less detectable than BUL pattern where $F^{-}$ removed was 162 mg. BUL = Bule Bule spring; FAP = fluorapatite; ICSD = Inorganic Crystal Structure Database; KYU = Ngarenanyuki borehole, Tanzania; OCP = octacalcium phosphate; XRD = X‐ray diffraction.

**Table S1.** Chemical synthesis reactions of (I) dicalcium phosphate dihydrate (DCPD) and (II) octacalcium phosphate (OCP); (III) Chemical transformation of OCP into fluorapatite (FAP) in presence of dissolved $F^{-}$

**Table S2**. Chemical composition of the tap water used for OCP synthesis

**Table S3.** Example behavior factors used in the analysis, and values.

## Supporting information

This article contains online‐only Supplemental Data.

Supporting information.

## Data Availability

We declare that all data are available by request to corresponding author Alfredo Idini (alfredo.idini@gmail.com).
