# Supplementary material for: Application of Octacalcium Phosphate with an Innovative Household‐scale Defluoridator Prototype and Behavioral Determinants of its Adoption in Rural Communities of the East African Rift Valley
Source: Integr Environ Assess Manag. 2020 May 1;16(6):856–70. doi: 10.1002/ieam.4262 (PMC7687156; doi:10.1002/ieam.4262)
Supplement: Supplementary file 1 — Supporting information. [file IEAM-16-856-s001.docx]

**SUPPLEMENTAL DATA**

**Application of octacalcium phosphate with an innovative household-scale defluoridator prototype and behavioral determinants of its adoption in rural communities of the East African Rift Valley**

Table S1. Chemical synthesis reactions of (I) dicalcium phosphate dihydrate (DCPD) and (II) octacalcium phosphate (OCP). (III) Chemical transformation of OCP into fluorapatite (FAP) in presence of dissolved F^-^.

|  |  |  |  |  |  |  |  |  |
| --- | --- | --- | --- | --- | --- | --- | --- | --- |
| Chemical reactions | |  |  |  |  |  |  |  |
|  |  |  |  |  |  |  |  |  |
| DCPD synthesis | | CaCO_3_ + H_3_PO_4_ + 2H_2_O → CaHPO_4_∙2H_2_O*_DCPD_* + CO_2_ + H_2_O | | | | |  | (I) |
|  |  |  |  |  |  |  |  |  |
| OCP synthesis | | CaHPO_4_∙2H_2_O ↔ 0.125Ca_8_(HPO_4_)_2_(PO_4_)_4_∙5H_2_O*_OCP_* + 0.25HPO_4_^2-^ + 1.375H_2_O + 0.5H^+^  (II) | | | | | | |
|  |  |  |  |  |  |  |  |  |
| OCP → FAP | | Ca_8_(HPO_4_)_2_(PO_4_)_4_∙5H_2_O*_OCP_* + 1.6F^-^ → 1.6Ca_5_(PO_4_)_3_F*_FAP_* + 1.2HPO_4_^2-^ + 5H_2_O + 0.8H^+^  (III) | | | | | | |
|  |  |  |  |  |  |  |  |  |

Table S2. Chemical composition of the tap water used for OCP synthesis.

|  |  |  |  |  |  |  |  |  |
| --- | --- | --- | --- | --- | --- | --- | --- | --- |
|  | pH | K^+^ | Na^+^ | Ca^2+^ | Mg^2+^ | SO_4_^2-^ | HCO_3_^-^ | F^-^ |
|  |  | mg/L | mg/L | mg/L | mg/L | mg/L | mg/L | mg/L |
| Tap Water | 8.03 | 1.5 | 17 | 23 | 7.4 | 22 | 96 | 0.15 |
|  |  |  |  |  |  |  |  |  |

| Table S3. Example behavior factors used in the analysis, and values | | |
| --- | --- | --- |
| Factors | Values | Example items |
| ***Risk Factors*** |  |  |
| Risk Vulnerability | 0-4 | How likely or unlikely is that you will develop dental fluorosis? |
| Risk Severity | 0-4 | If I had dental fluorosis this would affect my health severely |
| Risk Knowledge | 0-1 | Boiling water before consuming it |
|  | 0-1 | Filtering water before consuming it |
|  | 0-1 | Brushing teeth |
| ***Attitude Factors*** |  |  |
| Overall Attitude | -2 - 2 | Drinking filtered water from this new filter will be good for my health |
|  | -2 - 2 | Drinking filtered water from this new filter will reduce my medical expenditures |
|  | -2 - 2 | Obtaining drinking filtered water from this new filter will be time consuming |
|  | -2 - 2 | Drinking filtered water from this new filter will taste better than the water that I usually drink |
|  | -2 – 2 | Drinking filtered water from this new filter will make me feel happy |
| Norms Descriptive | -2 - 2 | If your community had the possibility of using this new filter system, most people will be consuming filtered drinking water. |
| Norms Injunctive | -2 - 2 | If your community had the possibility of using this new filter system, most of my neighbors will think that I should consume filtered drinking water |
| Norms Personal | -2 - 2 | If your community had the possibility of using this new filter system, I would feel a strong personal obligation to consume filtered drinking water |
| *Ability factor* | -2 - 2 | I believe I will have the ability to use this new filter system regularly |
| *Self-regulation factors* |  |  |
| Self-regulation Action planning | 0-4 | I would have a detailed daily plan on how to use this new filter system |
| Self-regulation Copying planning | 0-4 | I would have a detailed plan on what to do if this new filter system breaks |
| Self-regulation Perceived habit | 0-4 | I feel that the use of this new filter system will become an ingrained habit |
| Self-regulation Commitment | 0-4 | I would feel committed to use this new filter system every day |
| Self-regulation Forgetting | 0-4 | I would never forget to prepare drinking water from this new filter system |


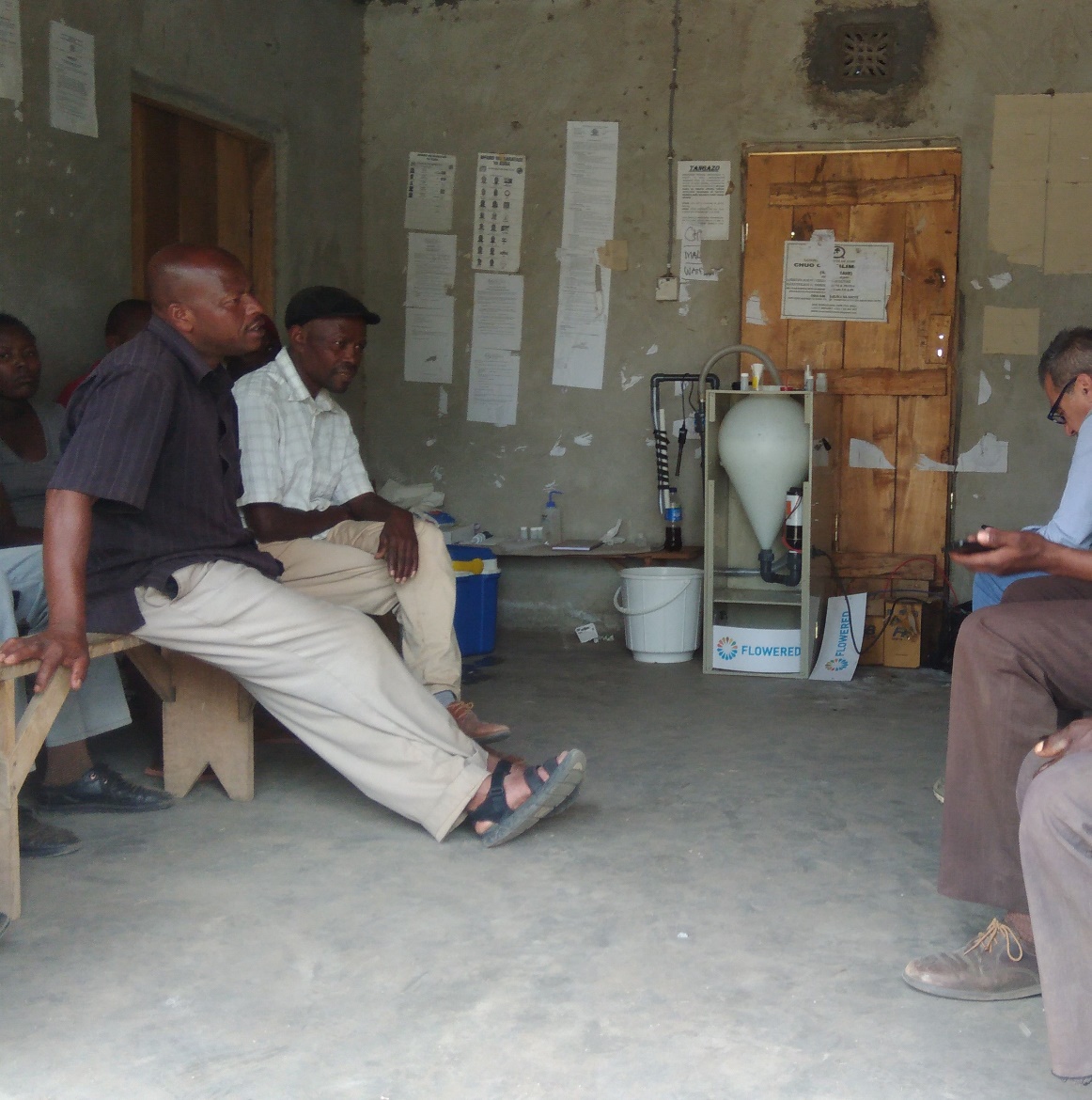


Figure S1. Site of defluoridation test and interview in the Uwiro village, Northern Tanzania.


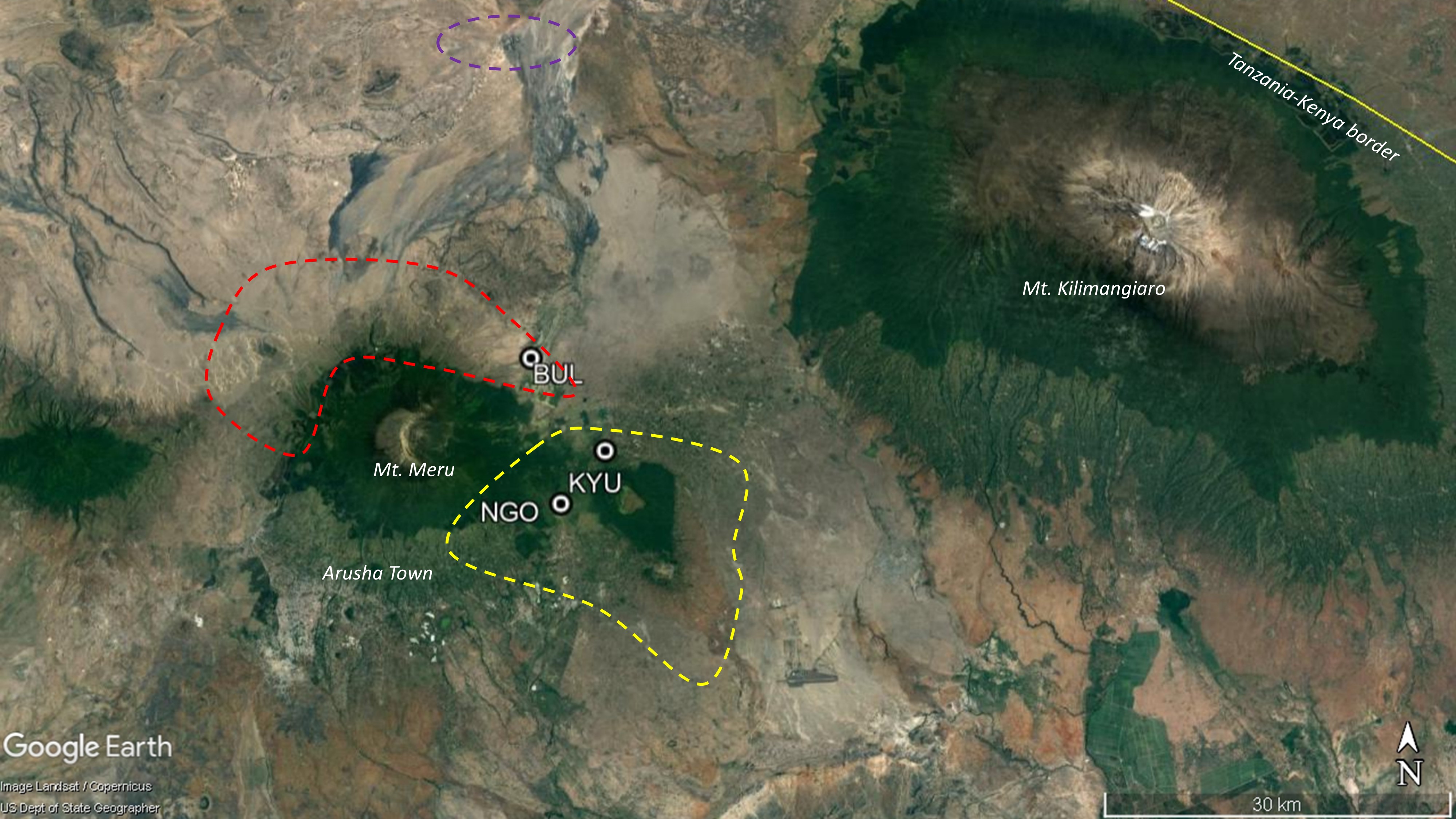


Figure S2. Location of water point source used for defluoridation test (dotted circle) and interview (purple line is around the Engutukoit village; red line is around Oldonyowas and Losinoni villages; yellow line is around Uwiro and Lemanda villages; Arusha Region, Northern Tanzania). Image © 2019 Digital Globe- Image 2019 CNES/Airbus.


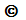

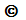

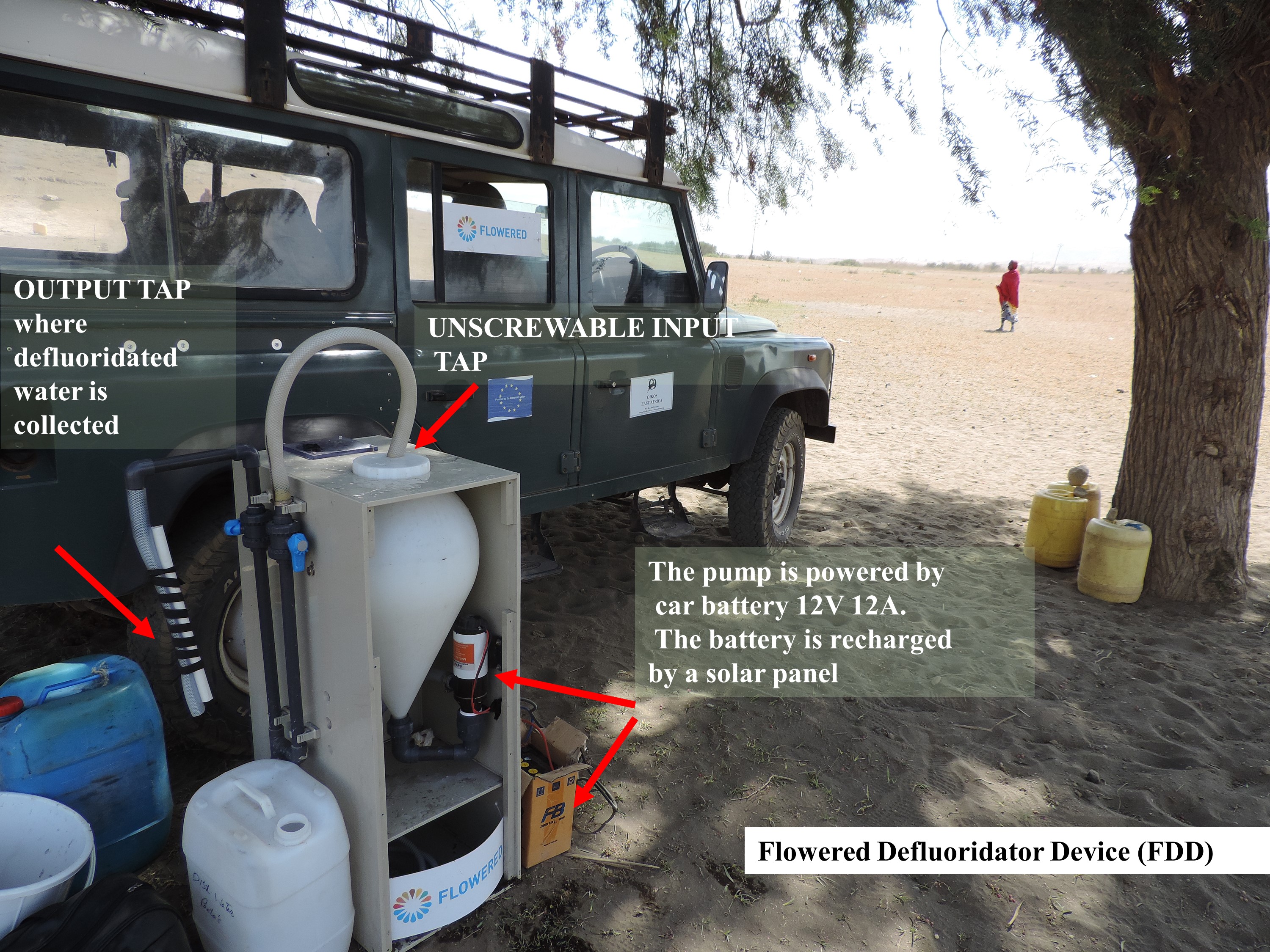


Figure S3. Configuration of the Flowered Defluoridator Device (FDD)


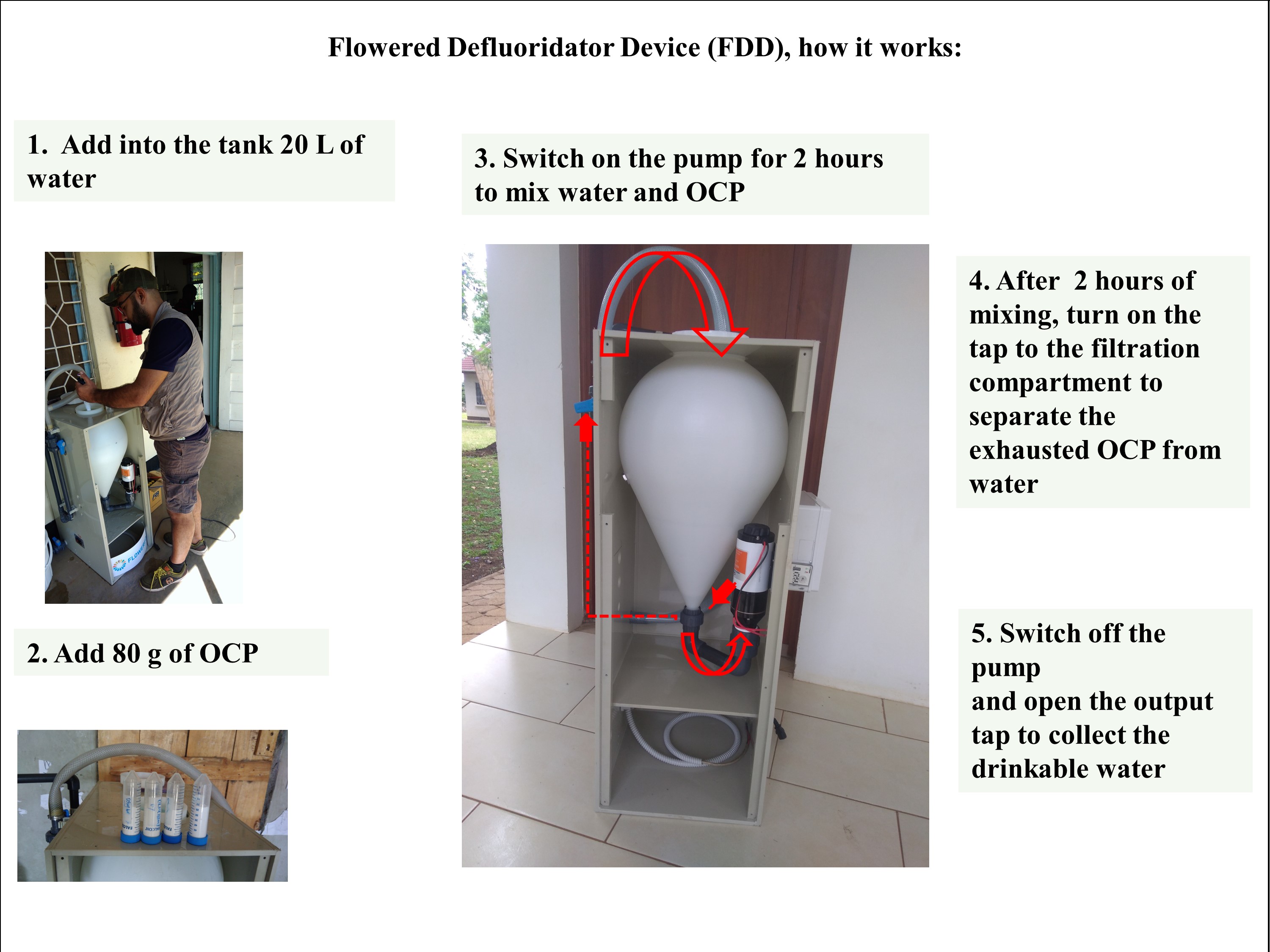


Figure S4. Step-by-step procedure for water treatment with the Flowered Defluoridator Device (FDD) and octacalcium phosphate powder (OCP)


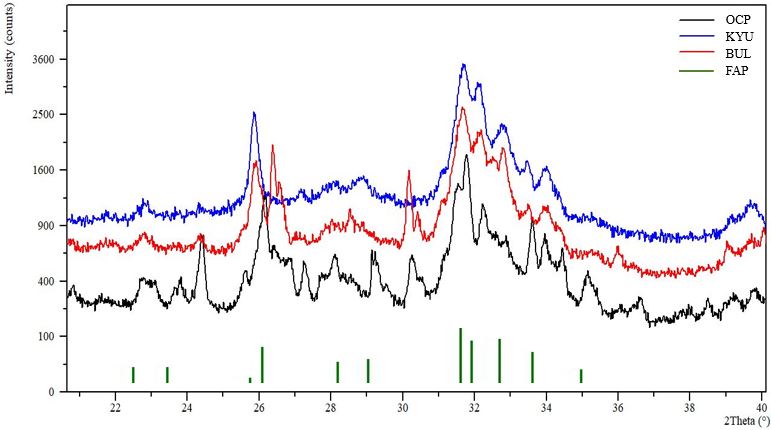


Figure S5. Difference of XRD pattern between OCP before tests (black pattern), solids collected after KYU (blue pattern) and BUL (red pattern) defluoridation tests and FAP reference ICSD pattern n. 00-015-0876 (green lines). In the pattern of KYU experiment, where 600 mg of F^-^ was removed from solution, the residual peak of OCP is less detectable than BUL pattern where F^-^ removed was 162 mg.
